# Calculation of the Stabilization Energies of Oxidatively Damaged Guanine Base Pairs with Guanine

**DOI:** 10.3390/molecules17066705

**Published:** 2012-06-01

**Authors:** Masayo Suzuki, Katsuhito Kino, Masayuki Morikawa, Takanobu Kobayashi, Rie Komori, Hiroshi Miyazawa

**Affiliations:** Kagawa School of Pharmaceutical Sciences, Tokushima Bunri University, 1314-1, Shido, Sanuki, Kagawa 769-2193, Japan

**Keywords:** oxidative guanine damage, base pair, hydrogen bond, stabilization energy

## Abstract

DNA is constantly exposed to endogenous and exogenous oxidative stresses. Damaged DNA can cause mutations, which may increase the risk of developing cancer and other diseases. G:C-C:G transversions are caused by various oxidative stresses. 2,2,4-Triamino-5(2*H*)-oxazolone (Oz), guanidinohydantoin (Gh)/iminoallantoin (Ia) and spiro-imino-dihydantoin (Sp) are known products of oxidative guanine damage. These damaged bases can base pair with guanine and cause G:C-C:G transversions. In this study, the stabilization energies of these bases paired with guanine were calculated *in vacuo* and in water. The calculated stabilization energies of the Ia:G base pairs were similar to that of the native C:G base pair, and both bases pairs have three hydrogen bonds. By contrast, the calculated stabilization energies of Gh:G, which form two hydrogen bonds, were lower than the Ia:G base pairs, suggesting that the stabilization energy depends on the number of hydrogen bonds. In addition, the Sp:G base pairs were less stable than the Ia:G base pairs. Furthermore, calculations showed that the Oz:G base pairs were less stable than the Ia:G, Gh:G and Sp:G base pairs, even though experimental results showed that incorporation of guanine opposite Oz is more efficient than that opposite Gh/Ia and Sp.

## 1. Introduction

**Scheme 1:**
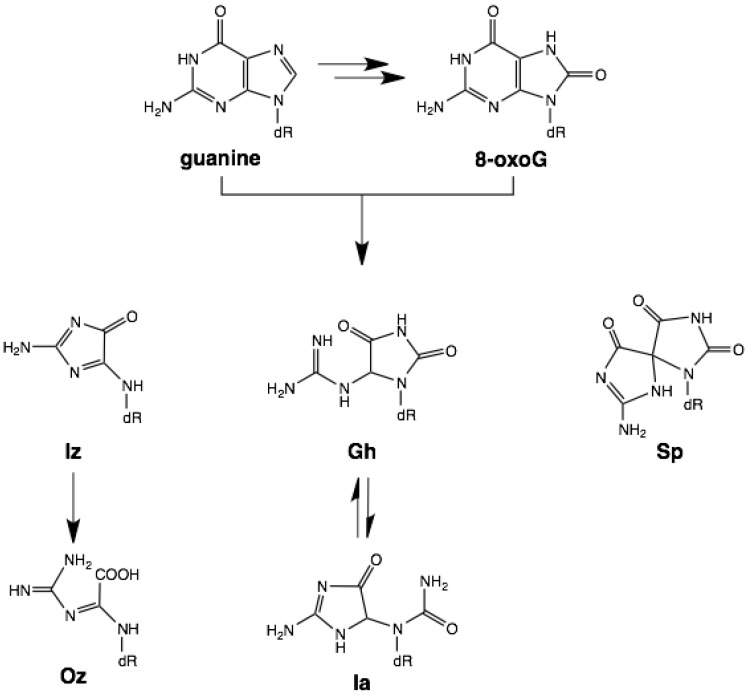
Oxidation products of guanine.

Genomic DNA is constantly assaulted by various endogenous and exogenous oxidative stresses. When the damage is not repaired, it can cause mutations which can lead to aging, neurological syndromes, carcinogenesis, and other diseases. Guanine has the lowest oxidation potential of the four bases and thus is highly sensitive to oxidative stresses in the DNA such as one-electron oxidation, singlet oxygen, and peroxynitrite exposure. G:C-T:A and G:C-C:G transversions are observed *in vivo*; for instance, G:C-T:A and G:C-C:G transversions caused by passive smoking are detected with high frequency in codons 12 and 13 of the *K-ras* gene [1]. Thus, investigations into the causes of G:C-T:A and G:C-C:G transversions will aid our understanding of the molecular mechanisms of DNA damage and repair. 8-Oxo-7,8-dihydroguanine (8-oxoG) is a representative guanine oxidation product formed under various oxidative conditions (Scheme 1). Since DNA polymerases insert adenine but not guanine opposite 8-oxoG, 8-oxoG:A base pairs cause G:C-T:A transversions [2]; that is, other forms of oxidative guanine damage appear to cause G:C-C:G transversions.

2,5-Diamino-4*H*-imidazol-4-one (Iz) can be formed from the oxidation of guanine and 8-oxoG (Scheme 1) [3]. The H-bonding donor and acceptor abilities of Iz are similar to those of cytosine, which can be paired with guanine. We previously reported that the calculated stabilization energy of the Iz:G base pair is similar to that of the C:G base pair [4]. These results confirm the observation that DNA polymerase I specifically incorporates guanine opposite Iz *in vitro* [5]. Thus, Iz may significantly cause G:C-C:G transversions. However, under physiological conditions Iz is hydrolyzed to 2,2,4-triamino-5(2*H*)-oxazolone (Oz) (Scheme 1) [3]. Primer extension by several DNA polymerases showed that guanine and/or adenine are incorporated opposite Oz [6,7]. We previously predicted that the calculated stabilization energy of Oz:G is larger than that of Oz:A, and that the Oz:G base pair has two hydrogen bonds and is planar [6].

Guanidinohydantoin (Gh) and spiroiminodihydantoin (Sp) are oxidation products of 8oxoG (Scheme 1). Gh is preferentially formed under acidic conditions, while Sp is formed under basic conditions [8,9,10,11]. It was previously shown that the Klenow fragment incorporates adenine and guanine opposite these lesions, and that the efficiency of guanine incorporation opposite Gh is higher than that opposite Sp [12]. Although Gh is known to isomerize to iminoallantoin (Ia) [13], it remains unclear which of these two isomers is predominant in DNA polymerization. We previously proposedIa:G and Sp:G base pairs [14], but the stabilization energies of these base pairs have not been calculated. Therefore, we estimated the stabilization energy of these base pairs by *ab initio* molecular orbital calculations using density functional theory (DFT) and a self-consistent reaction field (SCRF) method (*ε* of water) at a level of 6-31G**, and compared these results with our calculations for the Oz:G base pair.

## 2. Results and Discussion

### 2.1. Optimization and Calculation of the Stabilization Energies of Ia:G Base Pairs

**Figure 1:**
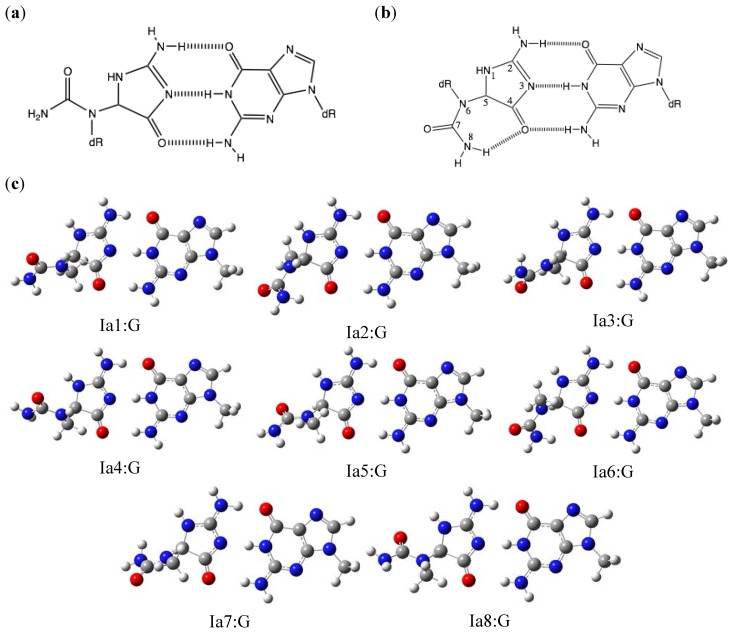
The Ia:G base pairs. (**a**) The proposed Ia:G base pair. (**b**) The proposed Ia:G base pairs and the associated hydrogen bonds. This numbering is the same as used in reference 13. (**c**) The geometries of Ia1:G-Ia8:G optimized by *ab initio* calculation. The stabilization energies are shown in 1.

Our previous study showed that Ia can pair with guanine, and that Ia:G can form three hydrogen bonds [14]. In this study, eight possible conformations of Ia (Ia1-Ia8) pairing with guanine were optimized by B3LYP/6-31G**. The geometries of Ia1:G-Ia8:G are shown in Figure 1c. The stabilization energies of these Ia:G base pairs were calculated and are shown in Table 1. The calculated stabilization energy of Ia1:G was 29.5 kcal/mol *in vacuo*, which is similar to that of the Watson-Crick C:G base pair (30.9 kcal/mol). This is not surprising, since both the Ia:G and C:G base pairs form three hydrogen bonds. In addition, the calculated stabilization energies of Ia3:G, Ia4:G, Ia5:G, Ia7:G and Ia8:G were also all 29.5 kcal/mol, indicating that moieties not involved in hydrogen bonding have little effect on the stabilization of the base pair. On the other hand, Ia2:G and Ia6:G contain an intramolecular hydrogen bond (O4-H8) (Figure 1b) and have stabilization energies of 28.7 kcal/mol. These two base pairs are destabilized compared to the other Ia:G base pairs by the delocalized electron density of O4. Thus, the stabilization energy of pairing with guanine appears to be reduced by a decrease in the electron density that contributes to pairing with guanine.

The stabilization energies of these eight base pairs in water were calculated as SCRF values. The stabilization energy of Ia1:G was the highest, at 24.1 kcal/mol, followed closely by Ia5:G at 24.0 kcal/mol. Ia1 has a (+)-*S* absolute configuration, and Ia5 has a (−)-*R* configuration [15]. Since the difference between Ia1 and Ia5 is only their *S* or *R* configuration, it is not surprising that Ia5:G has almost the same stability as Ia1:G.

**Table 1:** Stabilization energies (kcal/mol) of base pairs, obtained from the B3LYP/6-31G**-optimized geometries *^a^*.

Base pair	Δ *E*^DFT^	Δ *E*^SCRF^	Base pair	Δ *E*^DFT^	Δ *E*^SCRF^
Ia1:G	29.5	24.1	Gh7:G	19.9	19.5
Ia2:G	28.7	19.3	Gh8:G	19.8	16.9
Ia3:G	29.5	17.0	Gh9:G	21.0	19.1
Ia4:G	29.5	23.5	Gh10:G	20.9	17.3
Ia5:G	29.5	24.0	Gh11:G	20.6	20.6
Ia6:G	28.7	19.7	Gh12:G	20.4	19.6
Ia7:G	29.5	18.1	Gh13:G	20.3	16.6
Ia8:G	29.5	22.6	Gh14:G	20.5	17.1
Gh1:G	21.0	18.9	Gh15:G	20.8	18.5
Gh2:G	20.9	16.7	Gh16:G	21.1	19.0
Gh3:G	20.5	21.4	Sp1:G	28.2	18.8
Gh4:G	20.4	19.5	Sp2:G	28.2	19.9
Gh5:G	20.4	18.2	Oz:G	20.7	16.3
Gh6:G	20.5	16.8			

*^a^*Δ*E*^DFT^, in vacuo; Δ*E*^SCRF^, SCRF = Dipole, dilectric = 78.39, in water.

The stabilization energies of Ia3:G, Ia4:G, Ia7:G and Ia8:G in water were lower than those of Ia1:G and Ia5:G. In the Onsanger reaction field model, the solute is placed in a spherical cavity, surrounded by water. The larger the cavity radius, the more energy is required to eliminate water from the cavity. Since the calculated energy is dependent on the size of the cavity, decreasing the cavity radius contributes to the stability of the base pair [16]. Comparison of the *S* conformation structures ranked the cavity radius in the order Ia3 > Ia4 > Ia1, whereas comparison of the cavity radius of the *R* configuration structures provided the order Ia7 > Ia8 > Ia5. Therefore, Ia1:G and Ia5:G are more stable than the other base pairs. These calculated results suggest that destabilization in water depends on the molecular size of the base pair.

### 2.2. Optimization and Calculation of the Stabilization Energies of Gh:G Base Pairs

**Figure 2:**
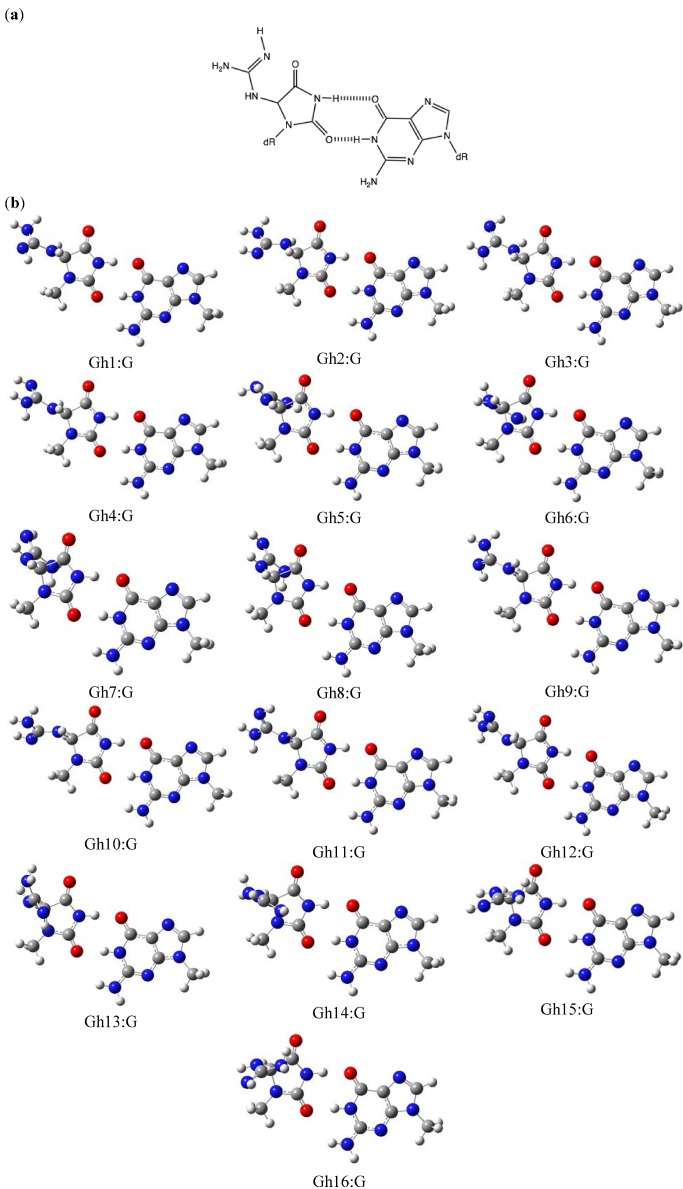
The Gh:G base pairs. (**a**) The proposed Gh:G base pair. (**b**) The geometries of Gh1:G-Gh16:G optimized by *ab initio* calculation. The stabilization energies are shown in 1.

Beckman* et al.* proposed that Gh:G forms two hydrogen bonds [17]. In aqueous solution, the diketo tautomers of Gh are estimated to be more stable than the enol tautomers [18]. Since Gh has three rotation axes and a chiral center, Gh has many structural conformations. Gh isomers 1-16 (Gh1-16) were optimized, and the stabilization energies of Gh1:G-Gh16:G were calculated and compared (Figure 2 and Table 1). Gh1-Gh8 have (−)-*S* absolute configurations, while Gh9-Gh16 have (+)-*R* configurations [19]. *In vacuo*, the calculated stabilization energies of Gh differed by ~2 kcal/mol. In contrast, in water the stabilization energy of the most stable base pair, Gh3:G, was 21.4 kcal/mol. Gh6:G was the most unstable (16.8 kcal/mol) of the (−)-*S* configuration Gh:G isomers. These results indicate that the difference in cavity radius, described above in the section dealing with Ia, has a greater influence on stability than does conformation. Thus, since Gh3:G has the smallest cavity radius of the Gh:G*S* configuration isomers, it has the highest stability. On the other hand, Gh11:G, with a stabilization energy of 20.6 kcal/mol, was the most stable of the (+)-*R* absolute configurations. The only difference between Gh3 and Gh11 is their *S* or *R* configuration, and it is not surprising that these represent the most stable of the isomers. Collectively, our calculated results show that the *S* configuration is more efficient at incorporating guanine than the *R* configuration. Since *S* and *R* diastereomers are interconvertible, these configurations coexist in solution [13,19]. Aller *et al.* previously proposed that the *S* configuration of Gh fits well within the DNA duplex and acts as a template. Therefore, our calculated results can explain the experimental data.

Both the *in vacuo* and in water calculated stabilization energies of Gh:G were lower than that for Ia:G. Our proposed Ia:G base pair has three hydrogen bonds. Taken together, the stabilization energy seems to depend on the number of hydrogen bonds. Thus, when guanine is inserted opposite Gh/Ia, Gh may tautomerize to Ia.

### 2.3. Optimization and Calculation of the Stabilization Energies of Sp:G Base Pairs

**Figure 3:**
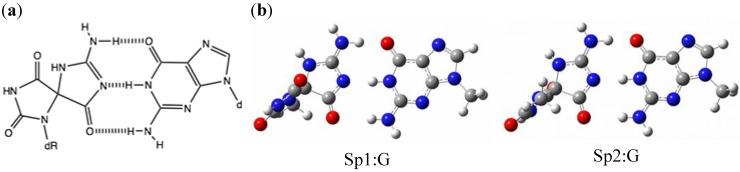
The Sp:G base pairs. (**a**) The proposed Sp:G base pair. (**b**) The geometries of Sp1:G and Sp2:G optimized by *ab initio* calculation. The stabilization energies are shown in 1.

We previously proposed that Sp can pair with guanine. Sp:G can form three hydrogen bonds, like Ia:G [14]. In aqueous solution, the triketo tautomers of Sp are estimated to be the most stable [18]. The absolute configurations of two Sp stereoisomers, Sp1 and Sp2, were previously determined: Sp1 is in the (−)-*S* configuration, and Sp2 is in the (+)-*R* configuration [15]. The calculated stabilization energies of Sp1:G and Sp2:G were 28.2 kcal/mol*in vacuo* (Figure 3), and in water Sp1:G and Sp2:G were 18.8 kcal/mol and 19.9 kcal/mol, respectively. As described in the section on Ia, since the cavity radius of Sp2:G is smaller than that of Sp1:G, Sp2:G is more stable than Sp1:G. Both *in vacuo* and in water, the calculated stabilization energies of Sp:G are less than that of Ia:G. In practice, the efficiency of guanine incorporation opposite Sp is less than that opposite Gh/Ia, and the block of translesion synthesis across Sp by DNA polymerase was much stronger compared to Gh/Ia [12]. Thus, our calculated results agree well with the previous experimental results.

### 2.4. Comparison of the Above Results with the Calculated Stabilization Energy of Oz:G Base Pairs

**Figure 4:**
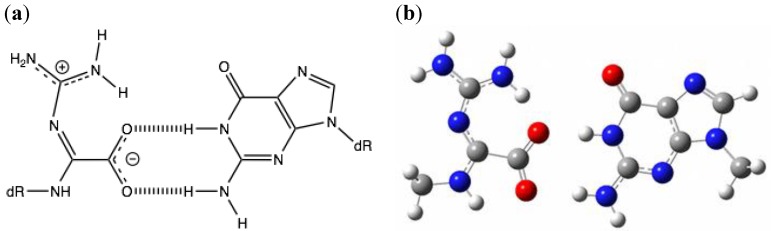
The Oz:G base pair. (**a**) The proposed Oz:G base pair; (**b**) The geometries of Oz:G optimized by *ab initio* calculation. The stabilization energies are shown in 1.

We recalculated the stabilization energy of the proposed Oz:G base pair and obtained a value of 20.3 kcal/mol *in vacuo* and 16.3 kcal/mol in water (Figure 4 and Table 1). These results indicate that the Oz:G base pair is less stable than Ia:G, probably due to the fact that Oz:G forms two hydrogen bonds whereas Ia:G forms three hydrogen bonds.

However, previous experimental results showed that the efficiency of translesion synthesis follows the order Oz > Gh/Ia > Sp [6,12]. Why, then, do the experimental results differ from the calculated results? It is important to note that the Oz:G base pair is planar, while Gh/Ia and Sp, which contain a sp^3^ carbon, are nonplanar. Stacking planar base pairs in DNA significantly enhances the stability of double stranded DNA, whereas pairing of nonplanar bases which contain a sp^3^ carbon causes destabilization of double-stranded DNA. For example, since the melting temperature of a Sp duplex is ~20 °C lower than that of a natural guanine duplex, Sp significantly lowers the thermal stability of the duplex [20]. Therefore, the experimental results might be explained by calculating the number of hydrogen bonds and accounting for the stacking effect.

In this study, we calculated the stabilization energy of oxidative guanine damage, in which CH_3_ was displaced instead of 2'-deoxyribose. Furthermore, the geometrical positioning of the C1'-carbons was not fixed during the optimization calculation. In addition, whereas the experimentally measured stabilization energy of the natural Watson-Crick C:G base pair is 21.0 kcal/mol [21], the DFT calculated value in water was 25.0 kcal/mol, 4.0 kcal/mol higher than the experimental value. This discrepancy is due to the calculation at the B3LYP/6-31G**. Since fixation of the C1' position and stacking are clearly important, we plan to recalculate with a different method in the future.

## 3. Experimental

All atoms were removed except for the oxidative guanine damages, guanine, the 2-deoxyribose C1' carbon and C1' H. Two H atoms were then attached to the C1' methine to complete the *N*-methylated nucleobases. The geometries were optimized at the B3LYP/6-31G** level using Gaussian 03 (Gaussian Inc., Wallingford, CT, USA) [22], and the stabilization energies *in vacuo* were calculated using optimized base pairs. Moreover, to estimate the energies in water, the SCRF values of these optimized base pairs were calculated using the Onsanger reaction field model and a dielectric constant of 78.39. The calculated heat of formation of the base pairs is defined in eq. (1): 


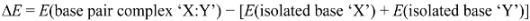
(1)

The optimized structures *in vacuo *were visualized with GaussView, as shown in Figure 1, Figure 2, Figure 3 and Figure 4.

## 4. Conclusions

The stabilization energies of Ia1:G and Ia5:G were higher than those of the other Ia:G base pairs in water, and were similar to that of the native C:G base pair (25.0 kcal/mol). In contrast, the highest stabilization energy of Gh:G, which has two hydrogen bonds, was 21.4 kcal/mol, significantly lower than that of Ia:G. Thus, the incorporation of Ia opposite G is more favorable that the incorporation of Gh opposite guanine. Sp2:G was slightly more stable than Sp1:G; the calculated stabilization energy of Sp2:G was 19.9 kcal/mol. Furthermore, the Sp:G base pair is less stable than the Ia:G base pairs. This difference in stability may explain why guanine incorporation opposite Gh/Ia is more efficient than opposite Sp. The calculated stabilization energy of the Oz:G base pair was 16.3 kcal/mol in water. Although experimental results show more effective incorporation of guanine opposite Oz compared to that opposite Gh/Ia and Sp, the Oz:G base pair is less stable than the Ia:G, Gh:G and Sp:G base pairs. In the future, calculations will need to consider not only the stabilization energy, but also the stacking effect and other physical properties. This will help address the current inconsistencies between experimental and calculated results.
